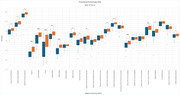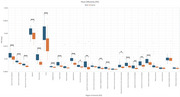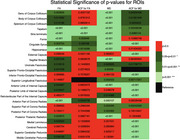# Microstructural White Matter Alterations in Alzheimer's Disease: A Diffusion Tensor Imaging Study

**DOI:** 10.1002/alz70856_105992

**Published:** 2026-01-11

**Authors:** Rufeyda Yagci, Tatjana Schmidt, Marcella Montagnese, Timothy Rittman

**Affiliations:** ^1^ Istanbul Medipol University, Istanbul, Istanbul, Turkey; ^2^ University of Cambridge, Cambridge, Cambridgeshire, United Kingdom; ^3^ Department of Clinical Neurosciences, University of Cambridge, Cambridge, Cambridgeshire, United Kingdom; ^4^ Cambridge University Hospitals NHS Foundation Trust, Cambridge, United Kingdom; ^5^ University of Cambridge, Cambridge, United Kingdom

## Abstract

**Background:**

White matter (WM) degeneration has been proposed as an early indicator of Alzheimer's disease (AD). Alterations in WM integrity, such as reductions in fractional anisotropy (FA) and increases in mean diffusivity (MD), may reflect the underlying pathological processes associated with AD. This study aims to investigate microstructural changes in AD utilizing Diffusion Tensor Imaging (DTI) and its applicability to a real world setting.

**Method:**

A total of 30 participants diagnosed with AD with a mean age of 68.5 ± 8.4 and 42 control participants with a mean age of 58.7 ± 7.7 were included, from the Quantitative MRI in NHS Memory Clinics (QMIN‐MC) study, recruited from NHS Memory Assessment Services. Diffusion‐weighted imaging (DWI) data were preprocessed using the Micapipe preprocessing pipeline to generate fractional anisotropy (FA) and mean diffusivity (MD) values. Twenty‐five regions of interest (ROIs) were selected from the JHU ICBM DTI‐81 Atlas and analyzed using FMRIB Software Library (FSL) tools.

**Results:**

We analyzed 25 ROIs to identify differences in FA and MD between AD and control groups. FA values showed significant differences in 17 regions (Figure 1), including the hippocampus, fornix, and cingulate gyrus, while MD values revealed significant differences in 18 regions (Figure 2). Normalization was performed using reference tracts considered least affected by age‐related changes: the superior longitudinal fasciculus (SLF) for FA and the superior fronto‐occipital fasciculus (SFO) for MD. After normalization, 6 regions showed significant differences for FA, and 18 regions for MD (Figure 3).

**Conclusion:**

Our findings support DTI measures as potential diagnostic tools for evaluation of AD patients as part of routine clinical practice. The involvement of key structures of the limbic system (hippocampus, fornix, and cingulate gyrus) along with white matter pathways such as the tapetum and stria terminalis provide evidence for the disruption of memory and executive processing, alongside inter‐hemispheric communication in Alzheimer's disease. Such insights provide a basis for further research into the microstructural changes underlying AD pathology and their real world diagnostic application.